# Two-dimensional single-cell patterning with one cell per well driven by surface acoustic waves

**DOI:** 10.1038/ncomms9686

**Published:** 2015-11-02

**Authors:** David J. Collins, Belinda Morahan, Jose Garcia-Bustos, Christian Doerig, Magdalena Plebanski, Adrian Neild

**Affiliations:** 1 Department of Mechanical and Aerospace Engineering, Monash University, Clayton, Victoria 3800, Australia; 2 Department of Microbiology, Infection and Immunity Program, Monash Biomedicine Discovery Institute, Monash University, Clayton, Victoria 3800, Australia; 3 Department of Immunology, Alfred Hospital Precinct, Monash University, Melbourne, Victoria 3004, Australia; 4 Therapeutics and Regenerative Division, Monash Institute of Medical Engineering, MIME, Monash University, Clayton, Victoria 3800, Australia

## Abstract

In single-cell analysis, cellular activity and parameters are assayed on an individual, rather than population-average basis. Essential to observing the activity of these cells over time is the ability to trap, pattern and retain them, for which previous single-cell-patterning work has principally made use of mechanical methods. While successful as a long-term cell-patterning strategy, these devices remain essentially single use. Here we introduce a new method for the patterning of multiple spatially separated single particles and cells using high-frequency acoustic fields with one cell per acoustic well. We characterize and demonstrate patterning for both a range of particle sizes and the capture and patterning of cells, including human lymphocytes and red blood cells infected by the malarial parasite *Plasmodium falciparum*. This ability is made possible by a hitherto unexplored regime where the acoustic wavelength is on the same order as the cell dimensions.

## Abstract (web summary)

Single cell trapping is very useful in biomedical applications, but to date this can only be done via mechanical traps. Here, Collins *et al.* use a two-dimensional acoustic field with wavelength comparable to cell size to pattern one cell per well in a microfluidic grid.

## Graphical abstract

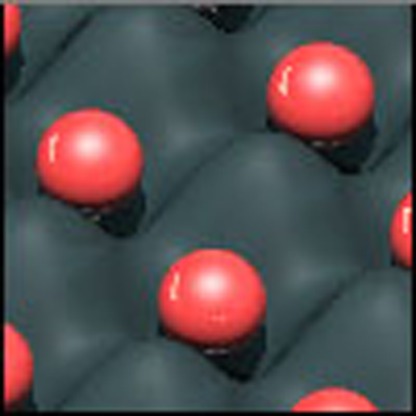

Trapping and patterning of cells is fundamental for purposes as varied as bioprinting^1^,^2^, cell–cell interaction studies^3^, drug development^4^ and single-cell analysis, a growing field of interest where heterogeneous cellular parameters are assessed on the basis of individual cells and their response to their local environment^5^,^6^. In cell studies, for example, constraining the location of cells according to defined positions permits the prolonged visualization of individual cellular development—potentially in the presence of modifying reagents—allowing the observation of phenomena that are averaged out in bulk cell culture. Restricting cellular patterns to two dimensions further aids in the visualization and study of individual cells, where the *en masse* trapping of 100–1,000 s of cells allows single-cell analysis on the scale of large populations^7^,^8^.

Microfluidic methods are a highly effective avenue for the patterning of single cells, where the dimensions of force gradients or physical features are, by necessity, on the same scale as individual cells (∼5–20 μm). Importantly, the distinction must be made between microfluidic methods that allow patterning of aggregates of cells or particles and those that enable this for individual ones; although patterning of cellular aggregates is useful for many applications, it is only through the spatial isolation of individual cells and the optical access that it affords that single-cell analysis is possible. A number of microfluidic techniques employ either hydrodynamic/mechanical methodologies or active forces to capture and pattern individual cells. Hydrodynamic methods serve to passively steer individual cells in a continuous flow to micro-patterned mechanical structures that spatially exclude more than a defined number of cells^9^,^10^,^11^,^12^,^13^. A major limitation of the mechanical trapping approach is that these devices are mostly single-use; when a cell is captured for a sufficient time it will adhere to the channel features and remain trapped. While this is sufficient for many long-term cell culture studies, for other applications such as the trapping and analysis of rare cells it is desirable to dictate both the time and duration of capture in addition to the location of cell trapping. A number of active techniques have been used for particle and cell manipulation and patterning, including optical^14^,^15^, magnetic^16^, electrical^17^ and acoustic^18^,^19^,^20^,^21^,^22^,^23^,^24^,^25^,^26^,^27^,^28^,^29^,^30^,^31^,^32^ forces, although these differ in their suitability to the patterning of individual, isolated cells. High-frequency acoustic forces—where periodically fluctuating pressure conditions result in time-averaged forces that push suspended matter towards acoustic nodes/antinodes—are generally biocompatible and have demonstrated potential for long-term cell observation^22^. This avoids problems such as the photobleaching of fluorescent enzymes and local heat stress associated with optical trapping, or the induction of strong electrical fields that can harm long-term cell viability in the case of dielectrophoretic forces.

Although acoustic fields have demonstrated patterning of particles and cells, for the most part the patterned cells form aggregates, rather than spatially isolated individual cells^19^,^33^. In this case it is still possible for a single cell to be individually trapped, although this is the outcome of using a low initial sample concentration, ultimately preventing the formation of relatively dense patterns otherwise available in hydrodynamic patterning methods^22^,^29^. There is, however, nothing about an acoustic field that inherently prevents the patterning of individual cells. By understanding the relevant forces in a high-density acoustic pattern and by imposing an acoustic field with a smaller wavelength than previously utilized, there is nothing to prevent the patterning of single cells in individual minimum-force locations. This physical regime, in which the particle or cell diameter *D* approaches the acoustic wavelength *λ*, has not been explored to date.

Here, we utilize surface acoustic waves (SAWs) at high frequency to create a two-dimensional (2D) acoustic force field with an inter-nodal spacing of the same order as the patterned cell dimensions. We hypothesize and experimentally verify that the patterning of spatially isolated cell patterns is feasible within a relatively narrow band of particle diameters as a ratio of the acoustic wavelength, a result of the combined and competing effects of the acoustic field and inter-particle interactions. When an acoustic field within this range is generated, both particles and cells are patterned in the 2D array of force potential minima with one cell per acoustic well (OCPW). Figure 1 shows schematics and a picture of the device that generates the acoustic field that is used to pattern particles and cells.

## Results

### Device principles and design

We generate a SAW by applying an a.c. voltage across a series of interdigital transducers (IDTs) that are patterned on a piezoelectric lithium niobate substrate; as with any piezoelectric material, a spatial gradient in electrical potential results in a mechanical displacement, which continues to propagate across the surface of that substrate—much as ripples in a pond—until dissipated by relaxation in the material itself or through a secondary material that the SAW couples into. SAW is a term describing a number of wavemodes whose displacements are concentrated at the substrate surface. SAW is uniquely suited to microfluidic manipulation of cells, with the capacity to locally generate an acoustic field of arbitrary aperture at a range of high frequencies (∼0.01–10 GHz) and subsequently small wavelengths (∼0.4–400 μm)^34^,^35^. Rayleigh-mode SAW, where the primary displacement in a single-material substrate is orthogonal to the surface and energy is transferred vertically into a fluid placed on top, is the most commonly used SAW mode for microfluidics applications^36^,^37^,^38^. Moreover, a SAW device is inherently planar, and is therefore simple to combine with other on-chip microfluidic features.

Applying the resonant frequency across the IDT electrodes so that the mechanical displacements from a finger pair, the minimum periodic unit of the IDT structure, reinforce one another results in a high-displacement SAW. The resonant frequency is given by *f*=*c*_s_/*λ*, where *c*_s_ is the speed of sound in the substrate and *λ* is the acoustic wavelength as determined by the spacing between adjacent IDT finger pairs. Despite the small surface displacements typical of MHz–GHz SAW, typically on the order of 0.1–10 nm, the resulting surface velocities are up to 1–10 ms^−1^, which drive up to MPa-order standing-wave pressures that can be used to capture particles and cells^39^. In addition, as a result of the surface-bound nature of the displacement, acoustic energy couples efficiently from the substrate surface to a fluid in contact with it. A standing wave in the horizontal plane of the fluid, necessary to trap particles, is induced through the intersection of two counter-propagating waves on the substrate surface. The addition of a channel roof in the path of this wave also results in a partial reflection of the propagating wave in the fluid, thus yielding a partial standing wave in the vertical plane that acts to constrain the motion of particles in this direction as well.

**Figure 1:**
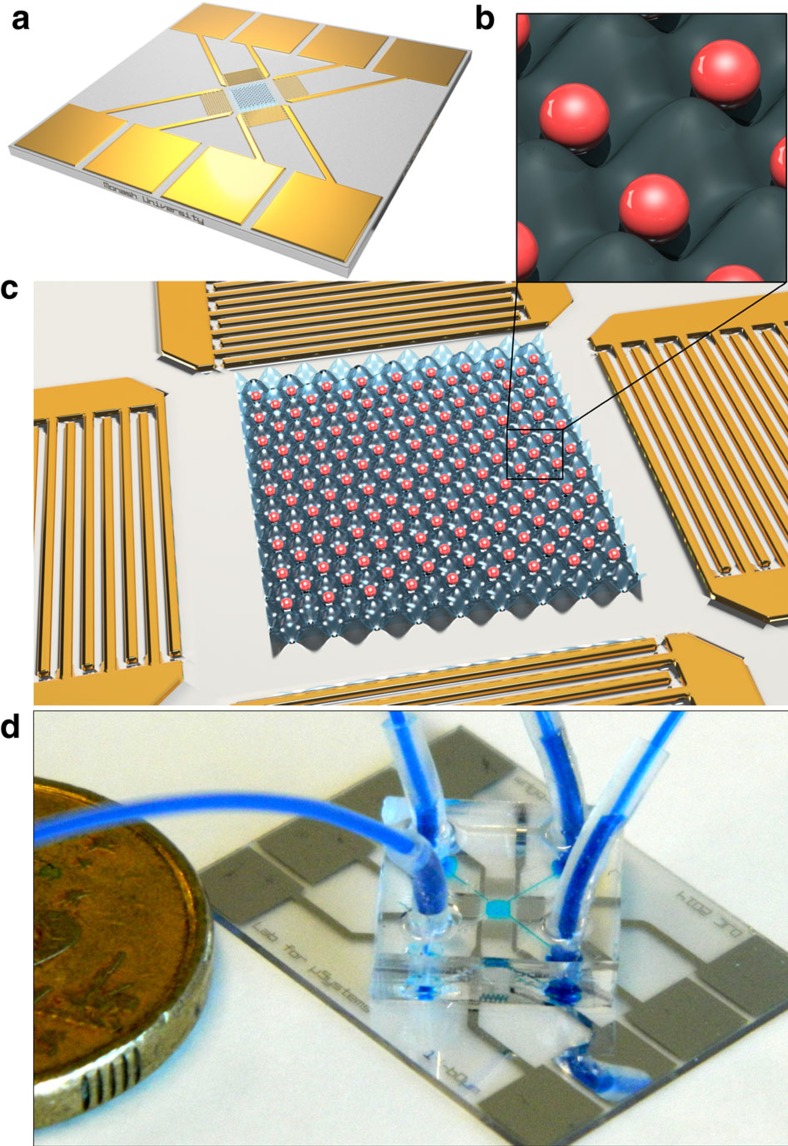
The design concept and example device used for OCPW patterning. (**a**) A OCPW device is comprised of a microfluidic chamber bonded to a piezoelectric substrate onto which interdigital transducers (IDTs) are patterned to generate four sets of intersecting surface acoustic waves (SAWs) that produce a high-frequency acoustic field in a coupled body of liquid. This field, represented by the 2D wavefield in **b** and **c** traps individual particles at nodal positions. (**d**) Here the chamber of the finished device is filled with blue dye for visualization, with an Australian $2 coin for scale. Adapted from ref. 70.

As the IDT spacing determines acoustic field's wavelength, different devices are used to access a range of OCPW-trapping regimes for multiple particle sizes, with 15, 18, 21, 23, 25 and 36 μm wavelengths, in addition to a wide-spectrum chirped device whose finger-pair wavelength and spacing varies from 14 to 60 μm, meaning that wavelengths in between these values can be accessed. See the Methods section for device fabrication details. A completed device is shown in Fig. 1d.

### Acoustic forces

In this section, we consider the forces experienced by a particle in a 2D acoustic field. We find that successful OCPW capture is not simply the imposition of a sufficiently small wavelength, below which single particles are patterned, but rather a relatively narrow band where the forces separating particles are dominant over those attracting them together. This interplay between these opposing forces, an understanding of which is necessary for single-particle patterning, has not been examined previously. Broadly put, a particle undergoes migration as a result of a time-averaged force acting on that particle when subject to an externally imposed acoustic field^40^. In a standing wave, this acoustic radiation force acts to move particles along a potential gradient towards the standing-wave node or antinode, depending on the respective densities and compressibilities of the particle and surrounding media; most types of cells and particles suspended in water, though, will migrate towards standing-wave nodal positions. However, in a system in which multiple particles are trapped and patterned simultaneously, each particle cannot be considered in isolation as they will also have an effect on the surrounding acoustic field. Here, the incoming wave, while driving particles towards the nodal positions, will also reflect and thus scatter a portion of the acoustic energy in the particle's vicinity, leading to secondary inter-particle forces (so-called Bjerknes forces)^41^,^42^, which may either be repulsive or attractive, though are more often attractive than repulsive for the typical media and particles/cells that are commonly used.

**Figure 2:**
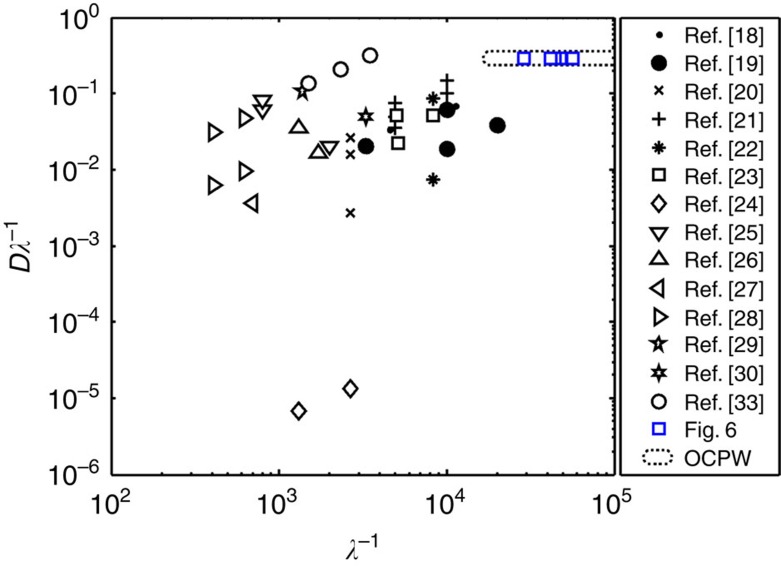
The patterning frequencies in comparison with previous acoustic trapping work. The bulk of research in acoustic microfluidics has focussed on the limiting case where the particle diameter is substantially less than the acoustic wavelength, with 
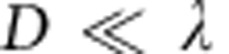
 and *f*∝*λ*^−1^, with typical frequencies of 1–50 MHz. Here, individual particles can be separated in individual acoustic force minima through the imposition of higher ratio of *D*/*λ*, a regime accessible due to the higher absolute frequencies utilized here, in the range of 100–230 MHz. The OCPW regime, which delineates the permissible *D*/*λ* and absolute values of *λ*, for trapping of cells (1 μm<*D*<20 μm) is encompassed by the dotted line shown.

**Figure 3:**
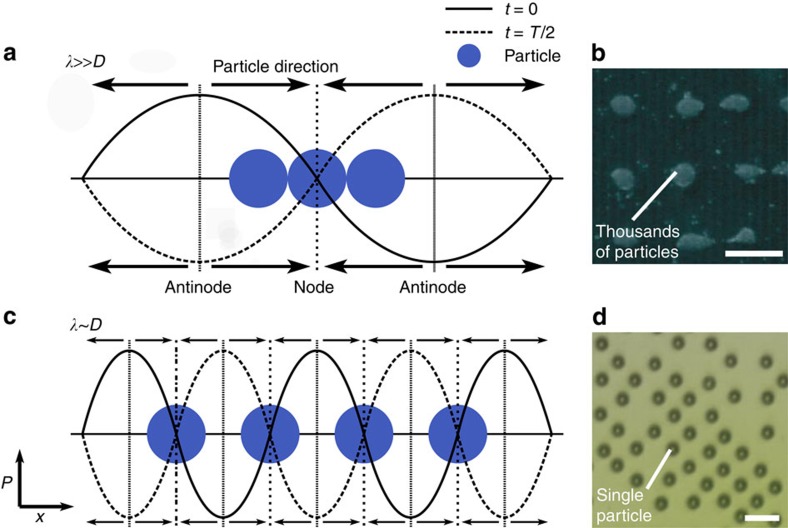
Comparison of the number of particles trapped by large and small acoustic wavelengths. A field with a characteristic wavelength on the order of the captured cell or particle diameter is required to pattern them individually. Looking at the simplified one-dimensional case, (**a**) given a relatively large wavelength (
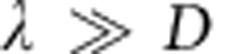
), multiple particles can cohabit in the same nodal position. (**c**) Although other considerations addressed in the text are important, individual particles can be spatially isolated given a small-enough acoustic wavelength proportional to the particle diameter (*λ*∼*D*). (**b**,**d**) The practical realization in a 2D field of these different regimes, patterning 10-μm particles in a 2.5-MHz field (reproduced with permission from ref. 26, Copyright 2007, Acoustical Society of America) and a 100-MHz one (from this work). Scale bar, 300 (**b**); 36 μm (**d**). Adapted from ref. 70.

The regime used for OCPW trapping, with particle dimensions *D* approaching that of *λ*, should be described in its relation to previous acoustic trapping work. In Fig. 2, it can be seen that substantially larger vales of *D*/*λ* and smaller absolute values of *λ*, to account for the small size of cells, are necessary for OCPW patterning. Through the imposition of particles at higher *D*/*λ*, only single particles can inhabit an individual nodal position due to steric considerations, in contrast to the previously demonstrated cases where particle aggregates form at these locations. The horizontal axis *λ*^−1^, underscores the substantially higher frequencies (on the order of *f*≳100 MHz, where *f*∝*λ*^−1^) than typically utilized that are required for the OCPW regime, with cell diameters on the order of ∼10 μm. Figure 3 explicitly illustrates these two different conditions, where 
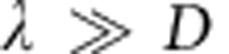
 (Fig. 3a,b) and *λ*∼*D* (Fig. 3c,d).

Unique to this work is the interaction between attractive interparticle forces and those confining particles to nodal positions in a manner that does not arise when 
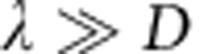
. In this case, the forces of acoustic radiation pressure and interparticle Bjerknes forces both have the same effect, namely particle aggregation at nodal locations (see Fig. 3a,b). Conversely, in the case where *λ*∼*D*, with a single particle per nodal position, these forces oppose each other. Unique to this study, it then becomes important to consider the force magnitudes of these counteracting effects to isolate individual particles. The equations used to determine the primary acoustic radiation force *F*_R_ and the inter-particle Bjerknes force *F*_B_ for a 2D standing wave are given by^42^


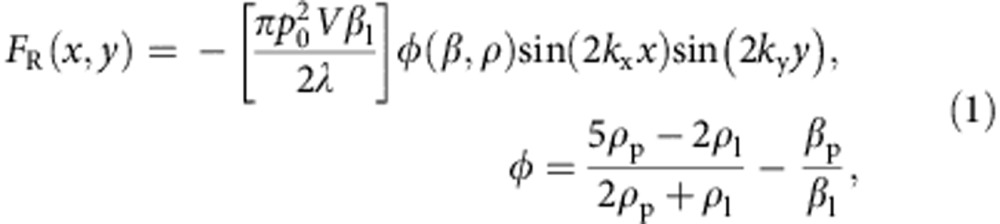


and


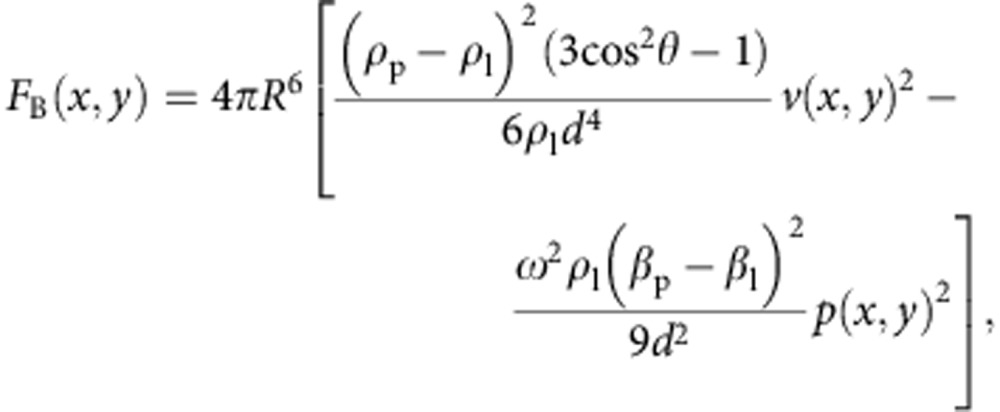


where *R* is the particle radius, *V* is the particle volume, *d* is the distance between particles, *θ* is the angle of the particle orientation relative to the wave propagation, *p*_0_ is the pressure field magnitude, *v*(*x*, *y*) and *p*(*x*, *y*) are the spatially varying velocity and pressure fields, respectively, and *ρ*_p_, *ρ*_l_, *β*_p_ and *β*_l_ are the densities and compressibilities of the particle and liquid, respectively. Both interparticle and standing-wave radiation forces, with *F*∝*R*^6^ and *F*∝*R*^3^, respectively, should be considered so as to select the range of acoustic wavelengths that form patterns with particles occupying adjacent nodal locations in the field, namely the ratio of *λ*/*D* in which single-particle patterning is possible. At the upper end of this range (that is, *λ*>4*D*), more than one particle can inhabit the same half wavelength. On the other hand, in the case of too small a wavelength, then particles at nodal positions in the field results in a *F*_B_ that is dominant over *F*_R_ if other particles are in the vicinity. This would result in particle aggregates, even though the *F*_R_ field itself remains periodic. These wavelength limits are explored for the polystyrene particles and red blood cells (RBCs) used in this work in Supplementary Fig. 1, where the magnitudes of the opposing forces |*F*_R_|, |*F*_B_| are considered and an estimate of the OCPW-trapping regime is explored. In addition, as periodic Rayleigh streaming vortices will occur as a result of nonlinear acoustic effects^43^,^44^,^45^,^46^, it is important to address their potential impact on particle trapping. Muller *et. al*.^47^ derived the expression for critical particle diameter, above which particle motion is dominated by the primary radiation force and below which the dominant mechanism is streaming-induced drag; for polystyrene particles (*ρ*_p_=1,030 kg m^−3^, *β*_p_=3.3^−10^ Pa^−1^) immersed in water at 100 Hz, this critical diameter is ∼200 nm, substantially smaller than those utilized here.

**Figure 4:**
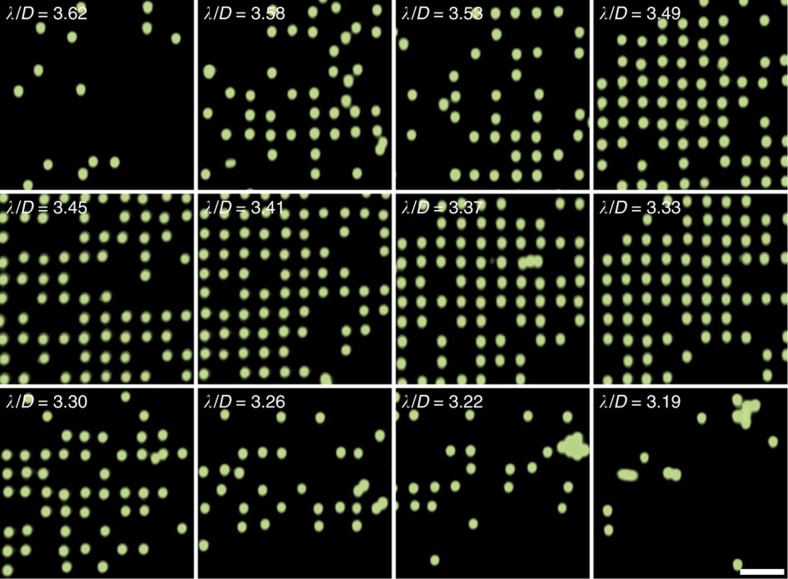
Effect of *λ*/*D* on particle patterning. As suggested here, there is a limited range of *λ*/*D* in which single particles can be patterned. The particle capture efficiency is shown for 6.1-μm polystyrene particles for a number of *λ*/*D* values, corresponding to odd-number frequencies in the range 163–185 MHz at an applied power of 0.38 W. Particle capture and patterning is limited outside of the range 3.2≥*λ*/*D*≥3.6. Fluorescent particle images (250 × 250 px) are obtained from the same central chamber region. Scale bar, 30 μm.

It is important to note that equations 1 and 2 have been analytically derived in the limiting case where 
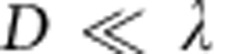
. In the case here, where *D*∼*λ*/3, the forces predicted by these equations are expected to deviate somewhat in both their scaling and absolute magnitude. There does not exist presently, however, analytical derivations of standing-wave acoustic force in the intermediate regime explored here, where the particle diameter is of the same order as the acoustic wavelength; most of the work in the acoustic microfluidic field has explored cases where 
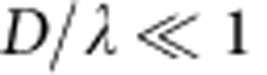
 (Fig. 2). Nor, indeed, is it a trivial task to develop analytical expressions in this regime, as the force scaling transitions in a nonlinear manner from *F*∝*R*^3^ to *F*∝*R*^2^ (ref. 48), though it should be possible for numerical methods with appropriate boundary conditions to bridge the gap between these two limiting cases. To obtain a preliminary empirical understanding in this intermediate regime, the observed particle-trapping regimes are compared with that expected from the theoretical predictions (Supplementary Fig. 1) in Fig. 4.

### 2D particle patterning

**Figure 5:**
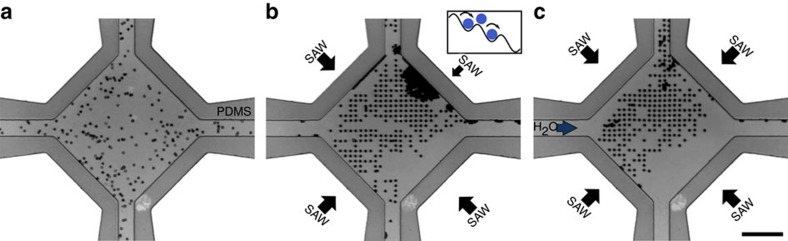
Methodology to increase seeding efficiency. (**a**) A solution of 6.1-μm particles suspended in water is (**b**) exposed to a *λ*=21-μm, 0.52-W-applied power, 171-MHz two-dimensional acoustic field biased in one direction. This biased field translates particles across the array through the force of the partial travelling wave thus created, effectively ‘bumping' particles along so that only one particle occupies a given nodal position (see inset), pushing most excess particles to the chamber wall in the direction of bias. (**c**) The excess particles can then be removed from the chamber by pumping fluid into the chamber, manipulated either by hand or a syringe pump. The majority of patterned single particles, however, remain trapped. Scale bar, 100 μm.

A major limitation of many acoustic patterning methods is that the effective seeding efficiency of the patterned grid is determined by the initial particle concentration; with too high a concentration, either multiple particles are trapped in the same intended space or will be located in between trapping locations. Too low initial concentrations lead to only sparse particle patterning. Figure 5 shows the methodology used to overcome this limitation, one which is made available by the ability to address individual SAW devices. The acoustic field can be biased in one direction by applying a reduced SAW amplitude to one set of transducers, thus creating a partial travelling wave that pushes suspended particles in the direction of the higher-amplitude wave propagation, while the 2D force potential minima ‘sieve' out individual particles. Here, the travelling wave force acts locally on particles against the partial standing-wave force, which is of sufficient magnitude and appropriate dimensions to retain individual particles but not groups of them, resulting in a seeded particle array with excess particles to the chamber wall. These latter particles can subsequently be removed with the application of a fluid flow, while single particles remain trapped in the acoustic array.

**Figure 6:**
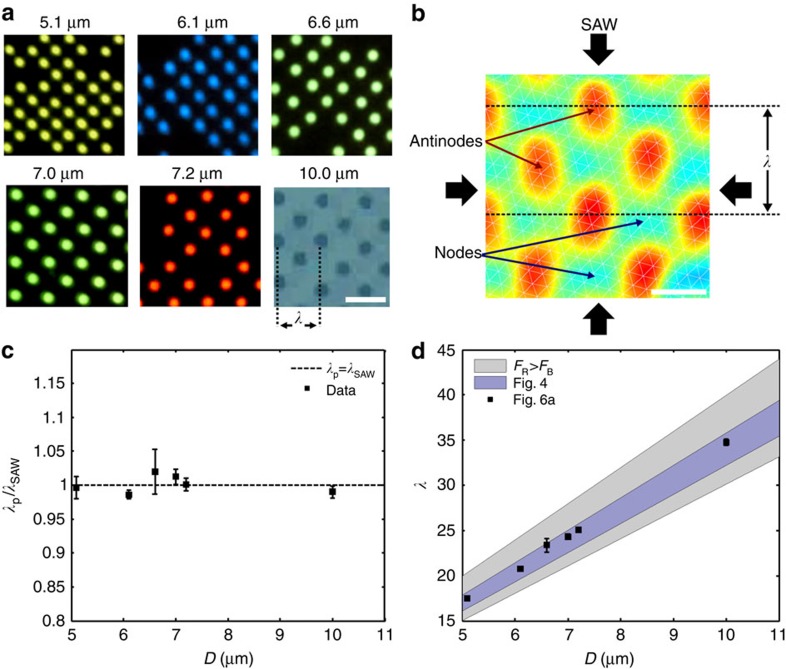
Characterization of the OCPW-patterning phenomena. (**a**) Spherical particles of essentially any size can be patterned in the two-dimensional acoustic grid, here showing the patterning of 5.1-, 6.1-, 7.0-, 7.2- and 10-μm polystyrene particles in acoustic fields of different wavelengths actuated by a 204-, 171-, 149-, 145- and 101-MHz (respectively) signal at 0.38 W. Scale bar, 30 μm. (**b**) The position of these particles is dictated by the nodal positions in the acoustic field, with particles migrating to the minimum displacement locations in the field. The image shown here is a map of the maximum surface displacement on a *λ*=30-μm device as measured by a laser doppler vibrometer. Scale bar, 15 μm. (**c**) As expected, the distance between the patterned particles in **a** is proportional to the wavelength of the acoustic field, with *λ*_p_/*λ*_SAW_=1, where *λ*_p_ is 2 × the distance between particles in the *x-* or *y*-directions. (**d**) The OCPW-trapping region (as measured by *λ*/*D*) scales linearly with particle diameter, although the range of these values is somewhat smaller than predicted by equations 1 and 2 (see Supplementary Fig. 1), suggesting that these equations may lose some predictive power as the particle dimensions approach that of the acoustic wavelength. Error bars in **c** and **d** denote one s.d. of the measured particle spacings in **a**. Adapted from ref. 70.

As discussed in the previous section, the acoustic wavelength used to pattern particles is important to operate in a regime where only one particle can inhabit a given space due to steric considerations, but particles are not so close that inter-particle forces dominate over that arising from standing waves. To characterize this system, polystyrene particles with *D*=5.1–10.0 μm are introduced into SAW devices whose wavelengths correspond to their pattering in Fig. 6a, where particles are patterned in the minimum-force potential (nodal) locations of the acoustic force field. A scan of the surface displacements on the SAW substrate is shown in Fig. 6b, where the time-averaged nodal (minimum displacement) and antinodal (maximum displacement) positions can be seen. The alignment of particles at the nodal positions in the horizontal plane has been explored previously by Collins *et al.*^49^. Figure 6c confirms the relationship between the observed inter-particle wavelength and the applied acoustic one, namely that *λ*_SAW_=*λ*_p_, the SAW and particle wavelength, respectively. Further, a linear relationship exists between the range of wavelengths that can be used to trap individual particles in square patterns and the dimensions of particles in those patterns; Fig. 6d shows the optimal trapping wavelength *λ*_opt_ for a range of particle diameters, showing *λ*_opt_∝*D*. Interestingly, particles and cells will self-segregate from cluster formations if the frequency is ramped up or down from to the optimal trapping wavelength; Supplementary Movie 1 shows the automatic grid arrangement and separation of lymphocyte cells (to be discussed in detail in the following section), as the frequency is continuously increased from 110 MHz (*λ*/*D*≈4) to 126 MHz (*λ*/*D*≈3.5).

The shaded blue region of Fig. 6d corresponds to the range of wavelengths that are empirically determined for polystyrene particle patterning, where Fig. 4 shows the effect that changing *λ*/*D* has on the seeding efficiency of the particle grid. Here, this range 3.2≥*λ*/*D*≥3.6 is somewhat smaller than the 3.0≥*λ*/*D*≥ 4.0 range suggested from the theoretical prediction where *F*_R_>*F*_B_ (Supplementary Fig. 1a), which corresponds to the shaded grey region in Fig. 6d. This is not entirely unexpected and suggests that the standing-wave force may drop off more quickly than the inter-particle Bjerknes force as the particle diameter approaches that of the wavelength. Indeed, this stands to reason whether one considers that the force acting on a particle is the sum total of all the force contributions from a spatially varying acoustic field; the maximum acoustic force occurs at the region of the highest force gradient. Because a particle size above the simplifying case where 
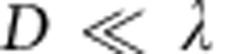
 will access a wider range of force gradients, all of which will be less than that at the nodal position, the net total of those forces will be less than that if only the force at the nodal position is considered. This concept, however, requires further exploration looking specifically at the magnitude of force generation for different *λ*/*D*.

The maximum theoretical width of the chamber in which particles can be patterned is ultimately determined by the relative magnitude of the local standing-wave and travelling-wave forces, and is discussed in Supplementary Note 1.

### Cell patterning

**Figure 7:**
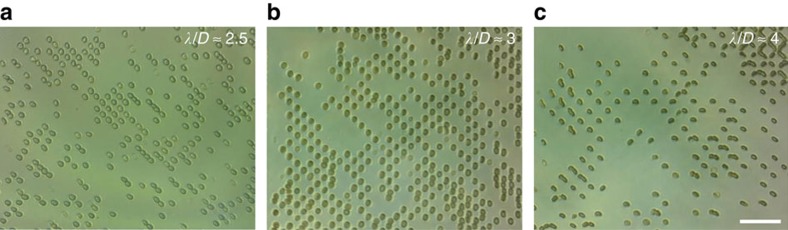
Red blood cell patterning. One cell per acoustic well (OCPW) can be produced for a wide range of wavelengths with respect to cell diameters *λ*/*D*. Here, red blood cells made semi-spherical by suspension in an hypotonic solution (0.8 × PBS) and patterned in a (from left to right); (**a**) 15 μm, (**b**) 18 μm and (**c**) 25 μm field (232, 200 and 144 MHz, respectively, at ≈0.5 W). While geometric considerations determine the lower limit of *λ*/*D*, where two cells cannot occupy the same space, the ability to pattern OCPW becomes strongly determined by the initial location of cells at the upper limit, theoretically at *λ*/*D*=4, above which multiple cells can co-locate in the same position. Scale bar, 50 μm.

Patterning with cells presents challenges and opportunities when compared with rigid synthetic particles. On one hand, the deformability of cells (especially those without cell walls) allows them to co-locate in acoustic wells for *λ*/*D* that would not be available to polystyrene spheres. However, as can be inferred from equation 2, their greater compressibility means that cells can typically be brought much closer together before Bjerknes forces dominate and bring them into direct contact. When suspended in isotonic conditions the RBC diameter is ∼7.5±0.5 μm (ref. 50), although with a thickness of only about 2 μm it is potentially more difficult to pattern these cells without instances of co-localization. Moreover, to better compare directly with spherical polystyrene particles and cell types that assume a mostly spherical shape in suspension, RBCs are suspended here in 0.8 × PBS, to inflate them to an oblate spheroid shape (diameter approximately 6–7 μm) as opposed to their normal biconcave disk shape, a transformation that has been explored previously^51^. Figure 7 shows the effective capture and patterning of individual RBCs over a range of *λ*/*D* wider than that for the polystyrene spheres in Fig. 6, with 2.5≥*λ*/*D*≥4.0. This is important for capturing large numbers of individual cells, given the natural variability in cell dimensions between cells at the same or different stages in development.

**Figure 8:**
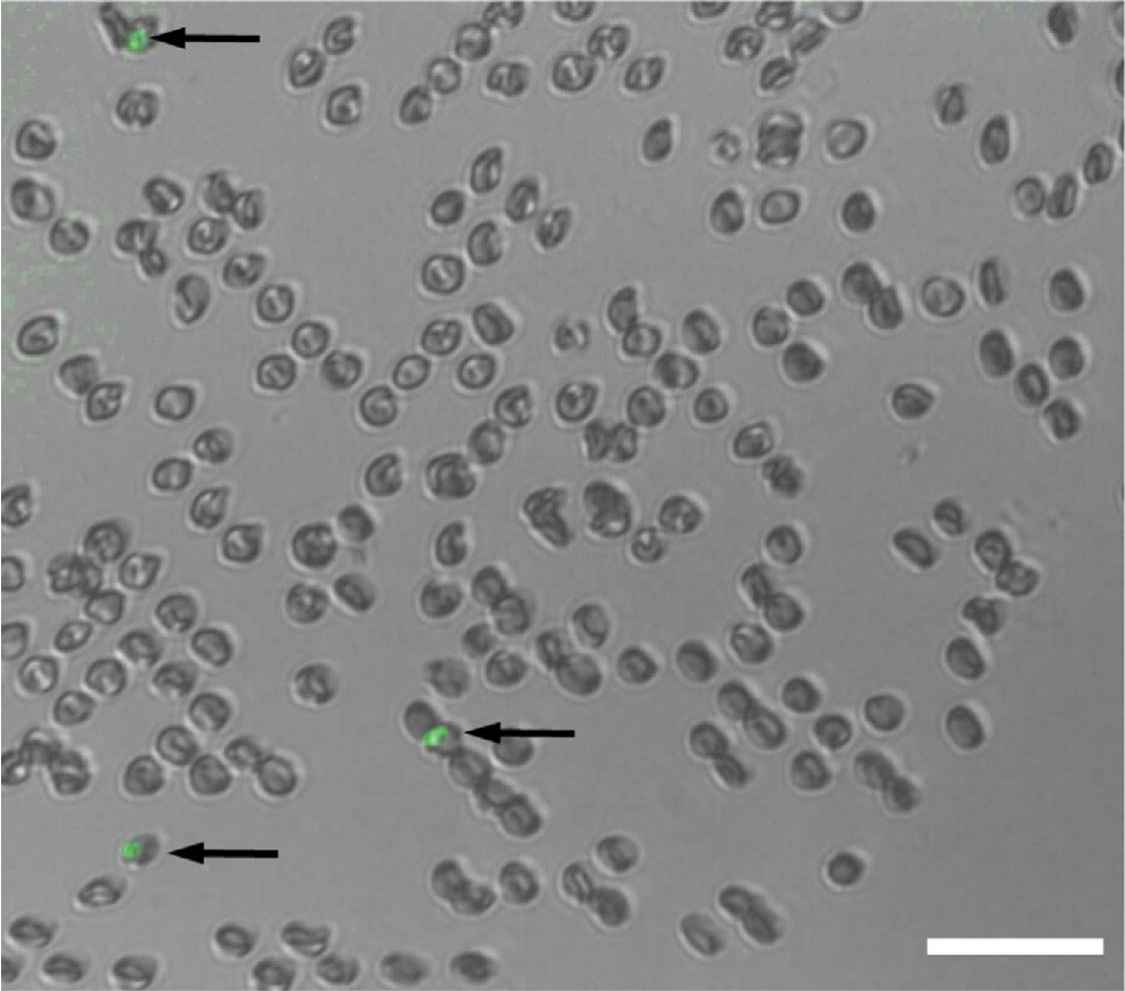
2D patterning allows for the spatial isolation and identification of individual cells. Here, a green fluorescent protein-expressing malarial parasite *Plasmodium falciparum* has infected a small proportion of a population of red blood cells (RBCs). In patterning this population in an 18-μm acoustic field (201 MHz at ≈0.5 W), infected RBCs can be independently visualized. Scale bar, 40 μm.

Even though RBCs are non-uniform in shape, unlike the case for most cells in suspension^52^, it is still possible to pattern non-spherical cells, in this case to produce 2D RBC cell patterns in their biconcave disk shape, despite the substantially smaller acoustic force that can be imparted (as *F*_R_ is proportional to the cell volume). To illustrate the potential of the OCPW system for studying low-frequency events, Fig. 8 shows captured RBCs, a fraction of which are parasitized by *Plasmodium falciparum*, here in the trophozoite phase. A strain of the parasite expressing green fluorescent protein is used to visualize and identify independent infected cells. Here, a 4.5-μm-high chamber is used to constrain cell movement in the vertical direction, in addition to the acoustic force field used in the horizontal plane; as opposed to the case with spherical RBCs patterned in a 10-μm chamber as in Fig. 7, highly deformable and thinner normal RBCs are more likely to co-locate in the same horizontal position. More broadly, this raises the point that the chamber height should be approximately on the order of the minimum cell dimensions, to avoid cells occupying multiple nodes in the vertical direction; this is less of a concern for polystyrene particles, however, where stronger scattering results in stronger particle–wall interactions that preferably favours certain trapping locations in the case of a non-symmetrical field in the vertical direction (with the piezoelectric substrate (LN) and polydimethylsiloxane (PDMS) as the chamber floor and ceiling). A consequence of using planar-shaped, highly deformable RBCs can be seen in some locations in Fig. 8, where a number of cells contact edge-to-edge despite being trapped. Here, it is hypothesized that these cells can reconfigure in response to the local pressure conditions, allowing small sections of cells to contact adjoining cells; this behaviour is more likely to occur in localized areas of the chamber, in which the SAW field is stronger in one direction due to non-uniformity in amplitude across the width of the IDTs (as noted in ref. 53). The larger diameter of flat RBCs may also contribute to the observed behaviour, where sphericalized RBCs will have a marginally smaller diameter (measured as ≈6.5 μm versus 7.5±0.5 μm for the literature value of flat RBCs^50^).

**Figure 9:**
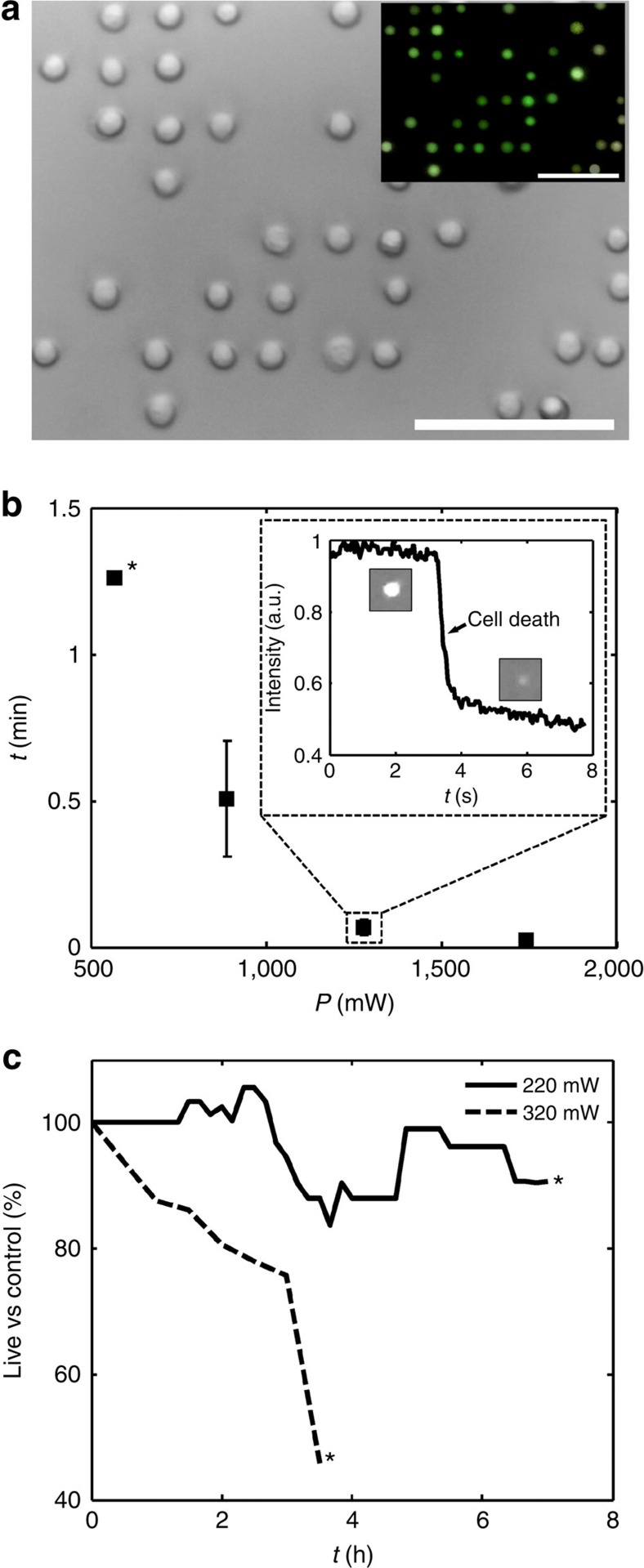
Lymphocyte trapping and viability. (**a**) Human peripheral blood mononuclear cell (PBMC) lymphocytes are simple to pattern as compared with RBCs due to their spherical shape. Inset shows a fluorescence image of carboxyfluorescein succinimidyl ester (CFSE)-infiltrated lymphocytes, a membrane leakage assay that demonstrates cell viability. Cells are patterned in a 126-MHz field from a continuous flow at 0.5 μl min^−1^ at 0.22 W, from an input concentration of 4,500 cells per μl to a local patterned concentration ∼100 × this. Supplementary Movie 2 shows individual cell capture in the array. Scale bar, 50 μm. (**b**) The power level limits the maximum exposure time, with the inset showing an example control-normalized fluorescence intensity measurement of membrane integrity loss (field applied at *t*=0). The time to lysis is strongly dependent on the applied power: although all continuously monitored cells (population (pop.) 11) are lysed within 49 s at 890 mW, only one is observed to do so at 570 mW within the ≈5-min period before photobleaching rendered the sample unusable, as denoted by (*). Error bars denote 1 s.d. for observed death times of 11 cells at 890/1,280/1,740 mW. (**c**) Below this threshold power (∼1 μW μm^−2^), cells can be trapped for increased periods with negligible effects on cell viability, here showing the long-term comparison of viable cells (pop. 50/48 cells for 220/320 mW studies) to a control (cells from same population incubated simultaneously in identical chamber without SAW, total pop. 57). Photobleaching of the sample (at *) is avoided for this duration through selective shuttering of the light source.

Any cell-trapping technique has the potential to impact cell viability, through induced shear strain or local heating, for example. The biocompatibility of MHz-order acoustic fields, important for long-term cell studies, has been the subject of recent study^23^,^32^,^54^,^55^,^56^, where viable cells have been trapped in nodal positions for durations on the scale of minutes to hours^18^,^22^,^57^,^58^. These studies are further validated in Fig. 9, which shows the patterning of human peripheral blood mononuclear cell lymphocytes tagged with carboxyfluorescein succinimidyl ester, a long-life cell membrane leakage assay used to track viability and movement of these cells (Fig. 9a inset). While acoustic power levels in excess of that required for stable trapping are sufficient to rapidly lyse these cells (Fig. 9b), cell lifespan rapidly improves for lower powers, establishing (for our system) an effective threshold of ∼570 mW, above which most lymphocytes will quickly lyse and below which cells can maintained in the acoustic field for long periods. Figure 9c shows that viability can be maintained over a period of hours, with ∼80% of cells alive after 2 h at 320 mW, and with negligible difference to the control sample at 220 mW, a power level shown in Fig. 9a and Supplementary Movie 2 to be perfectly capable of capturing the cells. These results are indicative of a typical human cell lymphocyte; however, different cell types will have different lysis threshold powers; individually patterned RBCs, which do not contain cell nuclei, are able to retain their morphologies over similarly long periods at powers of 500 mW (the power used in Figs 7 and 8).

**Figure 10:**
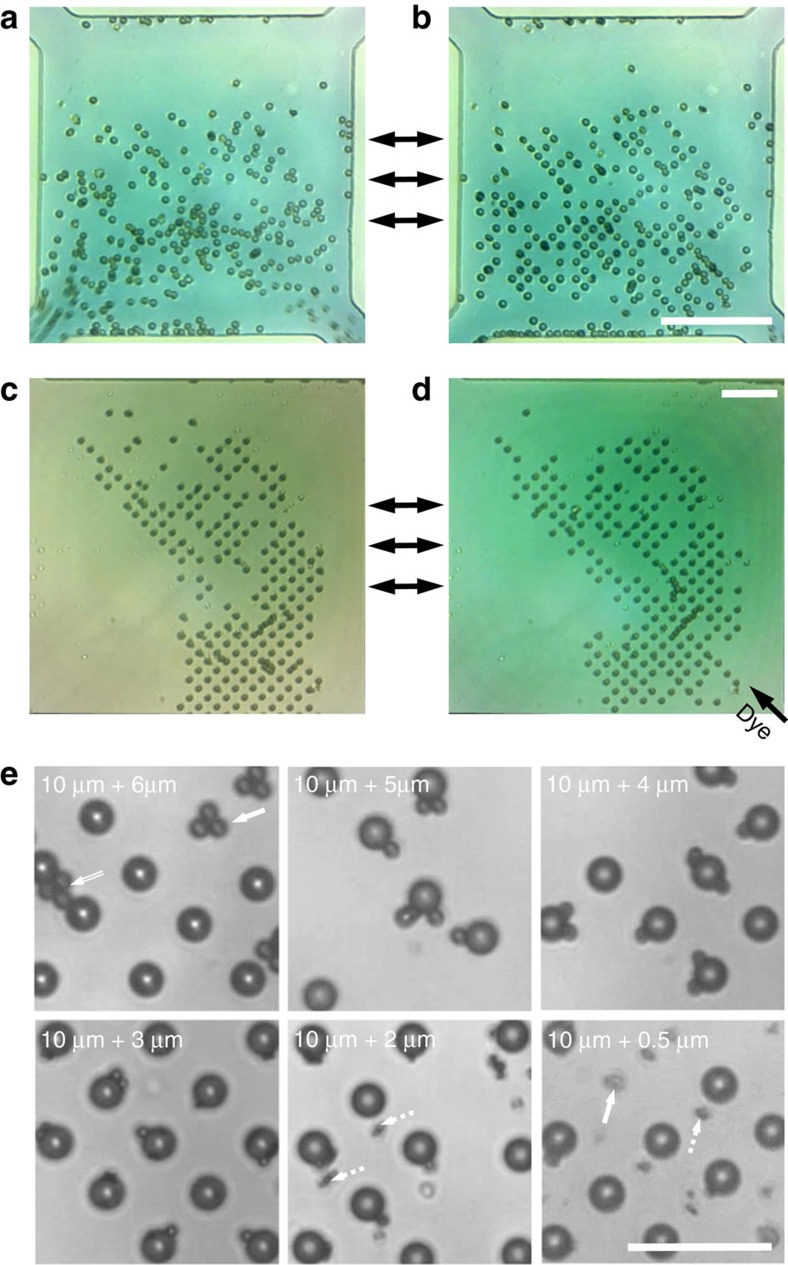
On-demand patterning with media exchange and particle co-location. (**a**) RBCs in a continuous flow can be (**b**) individually patterned and held for an arbitrary length of time through the imposition of an externally applied acoustic field (229 MHz at 0.25 W). The ability to hold particles and cells in place can further be used for applications such as media exchange, where **c** and **d** show the same trapped 6.1-μm particle grid suspended in water with a dye solution injected from a separate inlet, although some reconfiguring occurs due to changing local flow conditions (171 MHz at 0.52 W). Arrows between panels (**a**/**b**) and (**c**/**d**) denote interchangeability between random/patterned cells and suspension in different media, respectively. Using a patterned array of 10-μm particles, smaller particles can be trapped and co-located in the same locations, useful for cell-pair interaction studies where there is a large size difference between cells. (**e**) The limits to this activity, as defined by the difference between the size of the larger and smaller particle. When the smaller particle is larger than ≈50% of the larger particle's size, particles can either bridge between trapping locations (double-lined arrow) or cluster in a nodal location independently (solid arrow). Below ≈30%, particles can access trapping locations near the chamber roof offset from the primary trapping locations (dashed arrows). (**a**–**e**) Scale bars, 50 μm.

Batch processing of cells is made possible as the acoustic field can be arbitrarily applied. This temporal control of the force field allows cells to be patterned, analysed and released. A short demonstration of this, where suspended RBCs are patterned and then released on-demand in a continuous flow is shown in Fig. 10a and Supplementary Movie 3. Figure 10b shows that this ability to hold particles and cells in a continuous flow can be extended to exchange of medium, either to clear metabolites or introduce reagents so cellular response can be assayed. Array filling is also possible in a continuous flow, as Supplementary Movie 2 demonstrates, here with individual human lymphocytes automatically captured in nodal locations.

The OCPW system can also be used for other activities that would be difficult or impractical to realize in the mechanical trapping platforms previously demonstrated^59^,^60^, where paired cells are of similar dimensions. Figure 10c explores the limits of co-locating particles with significantly different sizes, where 10-μm particles are patterned in the conventional 2D grid and where smaller 3–5 μm particles (∼30–50% of the larger particle size) are preferentially retained and co-located in the same positions due to both inter-particle and primary radiation forces. Outside of this range, particles will either preferentially form clusters or trap in secondary locations offset to the primary field near the chamber extremes. Although these secondary locations are not the focus of this work, it is thought that the differential effect acoustic streaming has on smaller particles plays a role in this separation, as explored elsewhere^41^,^61^. This ability has the potential to be used in the study of single-pair bacterial/eukaryotic cell interactions, as a single-cell pairing extension of *en masse* cell culture work in the field^62^.

Finally, the range of patternable *λ*/*D* values gives an indication of the required monodispersity of patterned population. For polystyrene particles, the range of 3.2≥*λ*/*D*≥3.6 corresponds to a range of particle diameters ±6% relative to the mean *D*_avg_ that are permissible; the particles used here meet this criteria, which have a manufacturer-supplied coefficient of variation ≈3%. Cells, for which similar trapping results can be obtained over a wider range of *λ*/*D*, can accommodate a similarly wider range of diameters; for the RBCs tested, the 2.5≥*λ*/*D*≥4.0 range corresponds to ∼*D*_avg_±20%, which allows even moderately polydisperse populations to be trapped with OCPW.

## Discussion

In summary, we demonstrate a technique for the active pattering of spatially isolated individual cells in an acoustic field defined in two dimensions, including a characterization of the regime where OCPW patterning is possible. This is realized through the previously unexplored imposition of an acoustic field whose wavelength is of the same order of the cell dimensions, where only one particle can inhabit a given nodal location due to steric constraints. Rayleigh-type SAW wavemodes are uniquely suited to operate in this regime, where high-frequency fields are used to generate wavelengths on the scale of microns. Moreover, SAW-driven OCPW patterning is a biocompatible technique for the observation of single cells over long time periods that, in contrast to hydrodynamic/mechanical cell-pattering methods, is capable of re-use and batch processing. This latter capability is especially useful for the analysis of rare cells that may only make up a fraction of the total cell population. By patterning cells in spatially distinct positions, individual cells can be optically analysed over long periods in isolation from each other, a difficult task in conventional cell culture. Through the process of spatially isolating individual cells and constraining their positions, the activity of individual cells can be assayed, as opposed to the case in conventional bulk cell culture where parameters are effectively averaged across an entire population. This is especially important in pathogen biology, where infectious agents such as *Mycobacterium tuberculosis* can express many different phenotypes in a given population, relevant to antibiotic resistance^63^.

The ratio of wavelengths to diameters is crucial to single-cell patterning. In contrast to previous work where the acoustic wavelength is an order of magnitude or more greater than the particle or cell dimensions, reducing the acoustic wavelength sufficiently allows the patterning of individual, rather than clumps of particles. It is found that this single-particle-patterning effect is only workable for a relatively narrowly defined band of *λ*/*D* ratios, however, due to the competing influence of the force generated from the acoustic standing-wave field and those resulting from inter-particle interactions. Encouragingly though, cells are far less sensitive to inter-nodal spacing than synthetic particles, where OCPW can be achieved for a substantially larger range of wavelengths. Here, individual RBCs are spaced between 7.5 and 12.5 μm from each other, opening the door for future cell studies where inter-cellular distance is an important parameter. In addition, as batch processing is possible through the tunable application of the acoustic field, hundreds or thousands of cells can be analysed in a short period of time in a repeated fashion. This makes this method suitable for the isolation and analysis of phenomena affecting a small fraction of a cell population. Although outside the scope of this particular work, future applications for the OCPW-patterning system include long-term capture and observation for drug discovery, where short- and long-term cellular response to effector molecules can be assessed.

## Methods

### Device fabrication and set-up

In this work, four sets of IDTs, consisting of a 200-nm-thick Al conductive layer deposited on a 7-nm-thick Cr adhesive layer, are patterned on a piezoelectric 128° Y-cut lithium niobate (LN, LiNbO_3_) substrate. LN is often used in SAW microfluidic applications due to its high coupling coefficient and resultantly large surface displacements. Further, 300 nm of SiO_2_ is deposited on the substrate to prevent corrosion of the IDTs and to enhance channel bonding. Four sets of transducers, each rotated 90° around the focal region from neighbouring sets, are used to generate a two-dimensional sound field in which particles are patterned. Importantly, each transducer's SAW propagation axis is offset 45° relative to the usually preferred *x*-propagation direction. This orientation is chosen because the coupling coefficient is equivalent in all directions symmetric about this axis of the crystal, so that an equivalent number of finger pairs are required to produce an equivalent amplitude wave for each of the four sets of IDTs, although the sound speed in this direction is marginally lower at this offset, with *c*_s_≈3,590 m s^−1^ instead of *c*_s_≈3,960 m s^−1^ in the *x*-direction^64^. A 480 × 480-μm polydimethylsiloxane chamber (280 × 280 μm in Supplementary Movie 2) is created using soft lithography from an etched silicon mould and bonded to the LiNbO_3_ device using plasma-activated surface treatment. This chamber width is smaller than the 750 μm IDT aperture to maximize the uniformity of the acoustic magnitude within the chamber. In addition, to maximize the acoustic energy transferred to the liquid in the chamber, rather than to the PDMS that comprises the chamber structure, the IDTs are completely enclosed in an air-filled chamber with only a 56-μm-wide PDMS partition separating the respective air and liquid sections. The device is then actuated in all four channels by a signal generator (Belektronig powerSAW f20 for particle experiments, Hameg 8134–3 with Amplifier Research 25A250A for cell experiments), with a biased field produced by selective attenuation (with a 5-kΩ variable resistor) with particle and cell patterns viewed through a fluorescent microscope (Olympus BX53). Fluorescent polystyrene particles, all with coefficient of variation ≈3%, are obtained from Magsphere, USA, and RBCs are obtained from an anonymous healthy donor. To help prevent particle adhesion to the chamber walls, polystyrene particles are suspended in a 0.2% solution of polyethylene glycol. Surface displacement measurements are made by applying an a.c. signal at 0.126 W (Rohde&Schwarz SMBV100A) and measuring the resultant surface velocities with a laser doppler vibrometer (Polytec UHF-120).

### *P. falciparum* parasite culture

RBCs are obtained from the Australian Red Cross and suspended in 1 × PBS. Hypertonic solution is made with 4:1 1 × PBS/1% polyethylene glycol solution to make the RBCs spheroid. The *P. falciparum* parasite strain used in this study is D10/ACP signal-green fluorescent protein tagged^65^ and cultured *in vitro*^66^. For analysis, the cultures are synchronised to ring stages using 5% sorbitol^67^ and collected 24 h later. Temperatures of 35±2 °C are maintained on the microfluidic chip via a thermoelectric device on which the OCPW system is mounted, where steady-state temperatures are typically achieved on the order of seconds^68^,^69^.

### Peripheral blood mononuclear cells

Peripheral blood mononuclear cells (PBMCs) are isolated from blood collected in EDTA vacutainer tubes (BD Biosciences, USA) from healthy volunteers at the Monash Precinct at the Alfred Hospital, Melbourne, Australia (Human Ethics Committee Approval 2007002182) via Ficoll density centrifugation (GE Healthcare, Sweden), and washed three times in Dulbecco's phosphate buffered saline (dPBS, Sigma-Aldrich, USA). PBMCs are then fluorescently labelled by incubation for 5 min at in 0.4 μM carboxyfluorescein succinimidyl ester in dPBS at 37 °C at 10^6^ cells per ml, followed by quenching in ice-cold PBS media supplemented with 10% FCS (P10). Cells are then washed at 4 °C by centrifugation with P10, and resuspended to a final concentration of 150 μl/10^6^ cells in P10 for staining for 15 min with an APC-Cy7-labelled monoclonal antibody against CD4 (anti-CD4)(BD Biosciences). After further washing, cells are resuspended in P10 and cell sorted using flow cytometry using forward scatter versus side scatter gating for resting lymphocytes followed by selection of double-stained APC-Cy7 anti-CD4 (650/785 nm excitation/emission) and carboxyfluorescein succinimidyl ester (488/525 nm excitation/emission)-positive cells on the FACSAria high-throughput platform. The flow-sorted cells are washed and resuspended at 4.5 × 10^6^ ml^−1^ in cold P10, and kept in at 4 °C until application to the SAW device.

## Additional information

**How to cite this article:** Collins, D. J. *et al.* Two-dimensional single-cell patterning with one cell per well driven by surface acoustic waves. *Nat. Commun.* 6:8686 doi: 10.1038/ncomms9686 (2015).

## Supplementary Material

Supplementary Information Supplementary Figures 1 and Supplementary Note 1

Supplementary Movie 1 Cells will self-segregate from cluster formations if the frequency is ramped up or down from to the optimal trapping wavelength. The automatic grid arrangement and separation of lymphocyte cells is seen as the as the frequency is continuously increased from 110 MHz (λ~D/4) to 126 MHz (λ~D/3.5).

Supplementary Movie 2 Array filling is possible in a continuous flow. Here individual human lymphocytes are automatically captured in nodal locations, where incoming cells displace single trapped cells if a given nodal position is already occupied (freq. = 126 MHz, 0.22 W).

Supplementary Movie 3 Temporal control of the force field allows cells to be patterned, analyzed and released. Here, suspended RBCs are patterned and then released on-demand in a continuous flow by periodic actuation of the acoustic force field (freq. = 229 MHz, 0.25 W).
