# Supplementary material for: Two-dimensional single-cell patterning with one cell per well driven by surface acoustic waves
Source: Nat Commun. 2015 Nov 2;6:8686. doi: 10.1038/ncomms9686 (PMC4659840; doi:10.1038/ncomms9686)
Supplement: Supplementary Information — Supplementary Figures 1 and Supplementary Note 1 [file ncomms9686-s1.pdf]

Supplementary Figure 1

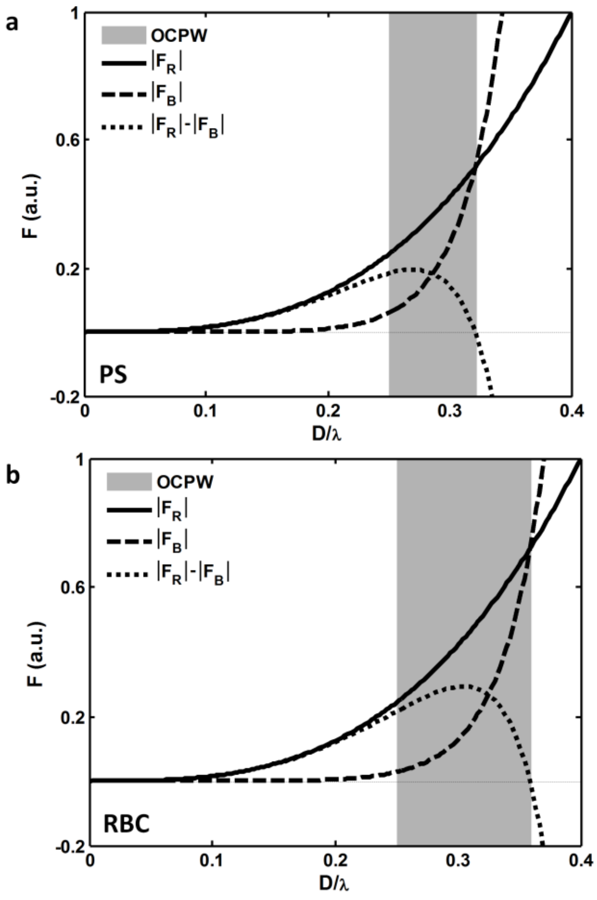

Supplementary Figure 1: This figure shows the theoretical regime in which individual particles can be trapped. Though the idea in Fig. 3 is conceptually simple, this belies the complex interplay between opposing forces that occurs in practice. Particle capture in a two-dimensional grid with particles in close proximity depends on the interaction between the acoustic wave radiation force F_R, which separates particles given a sufficiently small acoustic wavelength, and the interparticle so-called Bjerknes force F_B, which in the case of (a) polystyrene and (b) red blood cells suspended in water, acts to bring particles together. The range of distances particles can be separated while reliably retaining only one particle per acoustic well is thus dictated by two hard boundaries: for $D/\lambda < 1/4$, two particles could conceivably fit in the space between successive particles antinodes, whereas when $F_B > F_R$, interparticle forces will dominate over those retaining them in their nodal positions, resulting in particle clumping. F_R and F_B are calculated using Eqns. 1 and 2, respectively using the following parameters: $\rho_{w,p,rbc}$ = 998,1050,1092 kgm^{-3}, $\beta_{w,p,rbc}$ = 4.58,3.3,3.48 Pa^{-1}, where the *w*, *p*, and *rbc* subscripts denote values for water, polystyrene and red blood cells, respectively.

Supplementary Note 1

The standing wave component serves to pattern cells in the nodal locations, while the traveling wave component arises from the attenuation of the SAW amplitude due to energy transfer into the fluid, whereby the traveling wave is strongest at the channel edges in the direction of the wavefront propagation and the standing wave is strongest at the center, where wavefront amplitudes emanating from each set of transducers is equal. While the standing wave force is uniformly stronger than the traveling wave force for microparticles smaller than the wavelength given equivalent wavefront velocities, attenuation in the system gives rise to a special case whereby the standing wave force components will be lower than those of the traveling wave near the channel edges, provided the chamber width is sufficiently large. The width of the region in the OCPW device where the standing wave force is dominant (and therefore particle trapping occurs) is given by $W_{\mathrm{OCPW}} = \lambda\ell \log_e(F_R/F_{tw})$, where attenuation length of $\ell \approx 10$ is the number of wavelengths at which the SAW amplitude decays to $1/e$ [45,46], and F_R and F_{tw} are the standing wave and traveling wave forces. Solving this using expressions for F_R and F_{tw} that allow these quantities to be compared (from Ref. [40]), the maximum trapping width for polystyrene particles is given by $W_{\mathrm{OCPW}} \approx 70\lambda$, and $W_{\mathrm{OCPW}} \approx 60\lambda$ for red blood cells where $\lambda = 3.5D$. None of the chamber widths used in this work exceed these theoretical limits for the wavelengths utilized, so that trapping is possible at any location in our chambers. These expressions, however, are useful in illustrating the maximum theoretical number of trapping locations possible (e.g., N = $(70*2)^2$ = 19600 for polystyrene particles).
